# Histological and Clinical Findings in Patients with Post-Transplantation and Classical Encapsulating Peritoneal Sclerosis: A European Multicenter Study

**DOI:** 10.1371/journal.pone.0106511

**Published:** 2014-08-29

**Authors:** Joerg Latus, Sayed M. Habib, Daniel Kitterer, Mario R. Korte, Christoph Ulmer, Peter Fritz, Simon Davies, Mark Lambie, M. Dominik Alscher, Michiel G. H. Betjes, Stephan Segerer, Niko Braun, on behalf of the European EPS study group

**Affiliations:** 1 Department of Internal Medicine, Division of Nephrology, Robert-Bosch-Hospital, Stuttgart, Germany; 2 Department of Internal Medicine, Division of Nephrology and Transplantation, Erasmus Medical Center, Rotterdam, The Netherlands; 3 Department of Internal Medicine, Division of Nephrology, Albert Schweitzer Hospital, Dordrecht, The Netherlands; 4 Department of General, Visceral and Trauma Surgery, Robert-Bosch-Hospital, Stuttgart, Germany; 5 Department of Diagnostic Medicine, Division of Pathology, Robert-Bosch Hospital, Stuttgart, Germany; 6 Institute for Science and Technology in Medicine, Keele University, Keele, United Kingdom; 7 Department of Nephrology, University Hospital of North Staffordshire, Stoke-on-Trent, United Kingdom; 8 Division of Nephrology, University Hospital, Zurich, Switzerland; Université Paris Descartes, France

## Abstract

**Background:**

Encapsulating peritoneal sclerosis (EPS) commonly presents after peritoneal dialysis has been stopped, either post-transplantation (PT-EPS) or after switching to hemodialysis (classical EPS, cEPS). The aim of the present study was to investigate whether PT-EPS and cEPS differ in morphology and clinical course.

**Methods:**

In this European multicenter study we included fifty-six EPS patients, retrospectively paired-matched for peritoneal dialysis (PD) duration. Twenty-eight patients developed EPS after renal transplantation, whereas the other twenty-eight patients were classical EPS patients. Demographic data, PD details, and course of disease were documented. Peritoneal biopsies of all patients were investigated using histological criteria.

**Results:**

Eighteen patients from the Netherlands and thirty-eight patients from Germany were included. Time on PD was 78(64–95) in the PT-EPS and 72(50–89) months in the cEPS group (p>0.05). There were no significant differences between the morphological findings of cEPS and PT-EPS. Podoplanin positive cells were a prominent feature in both groups, but with a similar distribution of the podoplanin patterns. Time between cessation of PD to the clinical diagnosis of EPS was significantly shorter in the PT-EPS group as compared to cEPS (4(2–9) months versus 23(7–24) months, p<0.001). Peritonitis rate was significantly higher in cEPS.

**Conclusions:**

In peritoneal biopsies PT-EPS and cEPS are not distinguishable by histomorphology and immunohistochemistry, which argues against different entities. The critical phase for PT-EPS is during the first year after transplantation and therefore earlier after PD cessation then in cEPS.

## Introduction

Prolonged time on peritoneal dialysis (PD) could be complicated by encapsulating peritoneal sclerosis (EPS), a rare but severe complication [1]–[6]. Nowadays, three diagnostic hallmarks are used, i.e. clinical symptoms, radiologic findings and macroscopical/histological criteria [7]–[9]. In 2000, the International Society for Peritoneal Dialysis (ISPD) defined EPS by clinical signs of abdominal pain, bowel obstruction or weight loss in late stages of the disease [10]. Vlijm et al. [11] and Tarzi et al. [12] published computed tomography (CT)-based scores to diagnose EPS by radiological findings. Several working groups studied histological findings in EPS. However, diagnostic criteria are not well defined [13]–[15]. Recently, we established a scoring system based on morphological and immunohistochemical features [8]. This study was performed to distinguish simple sclerosis from EPS, more than 20 histological findings were studied and described.

Several risk factors for development of EPS have been reported. The risk of EPS increases with longer time on PD. Additionally younger age, glucose load, peritonitis rate, and cessation of PD are factors illustrated in some studies [5], [16], [17]. EPS may occur when patients are on dialysis (classical EPS, cEPS) or after undergoing a kidney transplantation (post-transplantation EPS, PT-EPS). The prevalence of PT-EPS has been reported to be between 1 and 3%. This presentation of EPS seems to occur shortly after kidney transplantation in former PD patients [18]–[21].

The pathophysiology of EPS is still unknown. The widely discussed second-hit theory assumes that the peritoneal membrane is “preconditioned” by the prolonged use of dialysis fluids resulting in a repair process with inflammation and fibrosis, so called simple fibrosis [21]–[23]. When the second-hit occurs, for example an inflammatory stimulus like bacterial peritonitis, or discontinuation of PD, EPS can develop [24]. There are several hypotheses how transplantation might act as a “second-hit”. These include discontinuation of peritoneal lavage of proinflammatory factors, direct apposition of damaged peritoneal membrane or, after successful kidney transplantation, concomitant use of profibrotic calcineurin inhibitors (CNIs) [7], [20], [21], [25]. Previously, Khanna et al. showed that both, Ciclosporine and Tacrolimus can enhance TGB-ß expression and subsequent fibrosis [26].

From a clinical point of view, both cEPS and PT-EPS are similar with regard to clinical presentation and radiological findings. However, post-transplantation EPS seems to be associated with less systemic inflammation at time of presentation and a better outcome [19], [21]. The purpose of the current analysis was to determine whether the morphological features of patients presenting with PT-EPS are different to cEPS, thus suggesting a different clinical entity. The clinical course following cessation of PD is also compared. For this purpose we combined peritoneal biopsies from two countries of an European consortium [7].

## Materials and Methods

### Study population

In the present study, 56 peritoneal biopsies were studied. All biopsies (n = 9) of PT-EPS cases in the biobank of the Dutch EPS registry and Rotterdam PA database (Netherlands) were selected and for each PT-EPS biopsy, one biopsy of a cEPS case was selected resulting in a total number of 18 biopsies [3]. Likewise, a total number of 38 biopsies (including 19 PT-EPS biopsies and 19 cEPS biopsies) were selected from the biobank of the Robert Bosch Hospital in Stuttgart (Germany).

In total, 28 biopsies from patients with PT-EPS were included in the present study. PT-EPS was defined as EPS in former PD patients undergoing a kidney transplantation, after which they developed EPS while having a functioning renal allograft. All 28 biopsies of cEPS patients were paired-matched for PD duration. After cessation of PD, none of the patients performed peritoneal lavage. Classical EPS was defined as EPS in patients who had been or were treated with PD without undergoing prior kidney transplantation.

For the diagnosis of EPS we used clinical criteria stated by Nakamoto et al. [27], radiological criteria by Vlijm et al. [11] and histological criteria by Braun and Honda et al [13], [28].

Data collection included demographic data, PD details at start of dialysis. The study protocol was approved by the medical ethics committee of Erasmus Medical Center and by the local ethics committee in Germany (#322/2009BO1, Eberhard-Karls University Tuebingen, Germany). All patients gave written informed consent before participating in the study.

### Peritoneal biopsies and analysis

Biopsies from the visceral peritoneum were formalin-fixed in 4% buffered formalin and paraffin-embedded following routine protocols [29]. All peritoneal biopsies were taken from patients at the time of catheter removal or during abdominal surgery (e.g. enterolysis, peritonectomy and enterolysis (PEEL)) following the protocol published by Williams et al. [30] in the time period from February 2002 to December 2012. Staining for podoplanin with the monoclonal antibody D2-40 has been used in several previous studies demonstrating the expression and pattern in EPS [8], [28], [31]. A monoclonal mouse antihuman podoplanin antibody (D2-40, DAKO, Baar, Switzerland) was used on all biopsies [8], [32]. A negative control specimen was created by omitting the primary antibody. Podoplanin was evaluated as either vascular or podoplanin avascular (0, 1, 2, 3). Furthermore, the histological description and pattern(s) of podoplanin-positive cells in peritoneal biopsies were investigated. The biopsies were separated into four groups (“low” podoplanin pattern, “organized” pattern, “diffuse” pattern and “mixed” pattern with features of both “organized” and “diffuse” patterns) [31].

From each slide hematoxylin and eosin staining was done for morphological analysis as previously described [8]: fibrosis: absent, 1–10%/low-power field (LPF), 11–50%/LPF, >51%/LPF (0, 1, 2, 3). Fibroblast-like cells (FLC): absent, 1/5 high-power fields (HPFs), 2–4/5 HPFs, >5/5 HPFs (0, 1, 2, 3); exudation: absent, 1 small area in 1 MPF, 1 area <50%/MPF, 1 area >50%/medium-power field (MPF) (0, 1, 2, 3); cellularity was evaluated as 0(1–2 nuclei/HPF), 1(3–5 nuclei/HPF) 2(6–20 nuclei/HPF) and 3(>20 nuclei/HPF); vessel density: absent, 1–5/HPF, 6–10/HPF, >10/HPF in the submesothelial cell layer (0, 1, 2, 3), acute inflammation (neutrophiles): absent, 1/HPF, 2–5/HPF, >5/HPF (0, 1, 2, 3); chronic inflammation (round cells): absent, 1–5/HPF, 6–20/HPF, >20/HPF (0, 1, 2, 3); hemorrhage: absent extravasal erythrocytes, 1 area <10%/5 LPF, 2+3 area/5 LPF or 1 area 11–30%/LPF, 4+5 area/5 LPF or 1 area >30%/LPF (0, 1, 2, 3); fibrin deposits: absent eosinophilic area, 1 area <5%/5 MPF, 1 area 6–20%/5 MPF, 1 area >20%/5 MPF (0, 1, 2, 3); presence of vasculopathy: thickening of vessel walls and/or inflammation of the vessel wall (0, 1); mesothelial denudation: no visible mesothelium (0, 1); presence of acellular areas (0, 1); presence of brown, probably iron deposits (0, 1); presence of blue, probably calcium deposits (0, 1), and osseous metaplasia (0, 1). FLC were defined as elongated cells, separated from vessel lumen with vesicular nucleus and one to three nucleoli. Acute inflammatory reaction was defined by the presence of neutrophilic granulocytes. Chronic inflammatory reaction was defined by the presence of round cells without taking into consideration further subclasses such as lymphocytes, plasma cells, monocytes and histiocytes. Furthermore thickness of the sub mesothelial cell (SMC) zone was measured as was descripted previously [30], [33], [34]. HPF = 0.26 mm^2^, MPF = 0.91 mm^2^, LPF = 3.2 mm^2^. Two experienced observer (one pathologist and one nephrologist) blinded to the specimen’s diagnosis evaluated each section.

### Statistical analysis

Continuous data are expressed as mean ± standard deviation (SD). Variables were classified as either binary (present or absent) or ordinal. The ordinal variables were discriminated as absent, low grade, moderate grade and high grade. We compared a four level classification system with a two level classification system. Each parameter was analyzed for its inter-observer variability. Comparisons between different disease groups were made using analysis of variances (ANOVA) and the Fisher-test. Statistical results with a p-value≤0.05 were considered as statistically significant.

## Results

**Figure 1:**
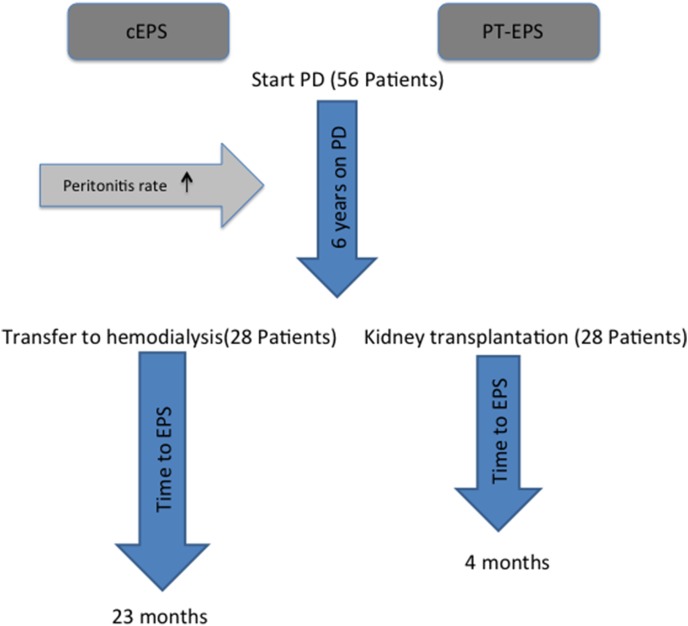
Schematic course of the studied patients. Fifty-six patients started PD. After a mean of approximately six years, twenty-eight patients were transferred to HD, whereas the other twenty-eight patients received a functioning renal allograft. Peritonitis rate was higher and the use of Icodextrin more common in the cEPS compared to the PT-EPS group. Time between transfer to hemodialysis and development of EPS was significantly longer, compared to time between transplantation and development of EPS (23(7–24) months vs. 4(2–9) months, p<0.001; cEPS classical EPS, PT-EPS post-transplantation EPS).

The baseline clinical characteristics of the study population are shown in table 1. A total of 56 EPS patients were included (28 PT-EPS and 28 cEPS patients, Figure 1). Eighteen patients from the Netherlands and thirty-eight patients from Germany were included. Time on PD was 78(64–95) months in the PT-EPS and 72(50–89) months in the cEPS group without a significant difference between the groups, indicating successful matching. In both groups, there were more female than male (p>0.05). Patients with cEPS demonstrated a significant higher rate of peritonitis episodes (most common organisms were *Staphylococcus aureus* followed by *coagulase negative Staphylococci* in both groups*)* and a more frequent use of Icodextrin (table 1).

**Table 1:** Clinical data of PT-EPS and cEPS patients; PD, peritoneal dialysis; EPS, encapsulating peritoneal sclerosis; PET, peritoneal equilibrium test, PDF, peritoneal dialysis fluid, *p<0.05, **p<0.001, ^#^median and interquartile range.

Variable	Post-transplantation EPS	Classical EPS
N	28	28
Age (years)^#^	52 (46–58)	55 (52–63)
Female/Male	17/11	21/7
CT diagnostic	28	28
Peritoneal thickening	13	12
Bowel dilatation	15	16
Calcification	7	9
Ascites	19	14
Clinical features		
*Bowel obstruction*		
Nausea and vomiting	23	22
Loss of appetite	18	15
Abdominal pain	28	26
Diarrhea	9	10
*Inflammation*		
Fever	10	7
PD details		
PD-duration at time of EPS diagnosis in months^#^	78 (64–95)	72 (50–89)
PET (switch to HD/NTx)	21	22
Low/low average	7	5
High average/high	14	17
Composition of PDF		
Neutral pH	6	10
Acidic pH	11	11
Both	8	3
N.D.	3	4
Icodextrin*	13/24	22/25
Peritonitis*	45 in 1990 months 1:44.2	103 in 1913 months 1:18.6
No peritonitis episodes	8/28	3/28
1–4 peritonitis episodes	19/28	17/28
>4 peritonitis episodes	1/28	8/28
Reason for cessation PD		
Peritonitis		10
Ultrafiltration failure		13
Technical failure		5
Age at time of NTx^#^	39 (32–47)	
Transfer to HD or NTx to diagnosis EPS (months)**^#^	4 (2–9)	23 (7–24)
Treatment after transplantation		
Tacrolimus	13	
Ciclosporin	9	
Both	6	
Follow-up		
Follow-up time (months)^#^	29 (22–74) months	31 (20–63) months
Alive/Dead	19/9	14/14

The time between cessation of PD and diagnosis of EPS was significantly longer in cEPS compared to time after transplantation in PT-EPS group (23(7–24) months vs. 4(2–9) months, p<0.001) (table 1 and figure 1). Time from onset of symptoms associated with EPS to requirement of surgery was 8(5–11) months in the PT-EPS and 5(4–8) months in the cEPS group (p = 0.7). Other parameters including outcome were not significantly different between the groups. All patients in the PT-EPS group were treated with CNIs as part of the transplant immunosuppressive protocol. Six out of twenty-eight patients in the PT-EPS group were exposed to both, Ciclosporin and Tacrolimus.

**Figure 2:**
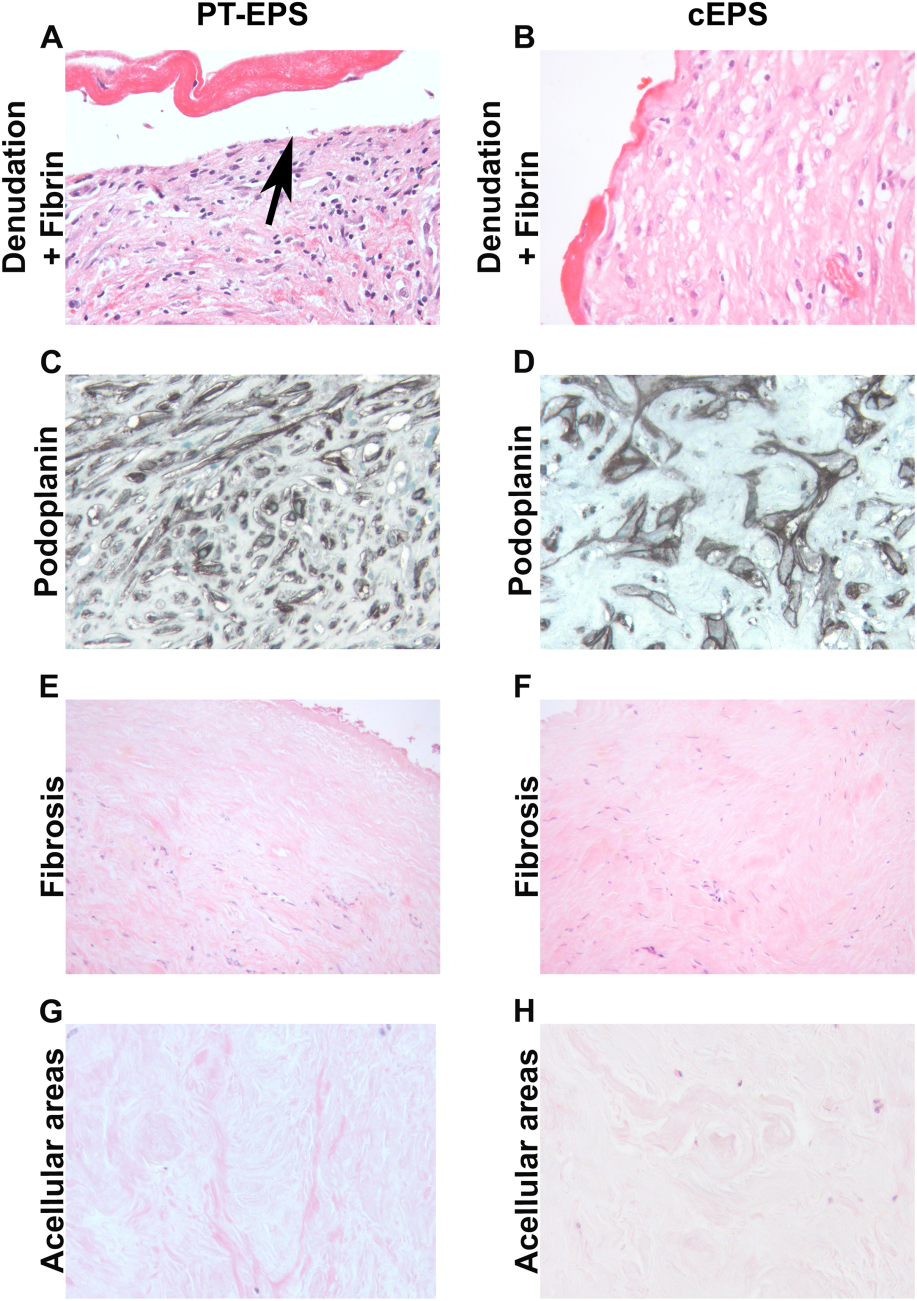
Morphologogical evaluation of peritoneal biopsies in PT-EPS and cEPS. Peritoneal biopsies were either stained with PAS (A, B, E–H) or by immunohistochemistry with a monoclonal antibody against podoplanin (D2-40, C, D, original magnifications X 400 in A–D, G, H, X200 in E, F). The morphological evaluation demonstrated similar degrees of denodation and fibrin deposition (A, B), podoplanin positive cells (C, D), fibrosis (E, F) and acellular ares (G, H). (left column post transplant EPS; PT-EPS, right column classical EPS, cEPS).

A detailed evaluation of the biopsies from 28 PT-EPS patients and of 28 patients with cEPS was performed (Table 2, Figure 2). Two observer blinded to the diagnosis studied the slides. Examples of the 17 parameters were illustrated in figure 2. As previously described the majority of EPS biopsies demonstrated severe fibrosis, accumulation of fibroblast like cells, mesothelial denudation, fibrin deposits and chronic inflammation (Table 2). The degree of fibrosis was measured and additionally analyzed semiquantitatively. Measurement of fibrosis revealed a fibrosis zone of 1369 µm [IQR 946–2551] in cEPS and 1690 µm [IQR 1356–2598] in PT-EPS (p = 0.17). Furthermore, podoplanin positive cells and podoplanin positive lymphatic vessels were prominent features (Figure 2).

**Table 2:** Histological findings in patients with post-transplantation EPS and classical EPS; Fibrosis (0, 1 vs. 2, 3), Fibroblast-like-cells (FLC) (0, 1 vs. 2, 3), Exudation (0, 1 vs. 2, 3), Mesothelial denudation (0 vs. 1), Acellular areas (0 vs. 1), Cellularity (0, 1 vs. 2, 3), Vessel density (0, 1 vs. 2, 3), Acute inflammation (0, 1 vs. 2, 3), Chronic inflammation (0, 1 vs. 2, 3), Vasculopathy (0 vs. 1), Hemorrhage (0, 1 vs. 2, 3), Fibrin deposits (0, 1 vs. 2, 3), Calcification (0 vs. 1), Iron deposits (0 vs. 1), Ossification (0 vs. 1), Podoplanin vascular (0, 1 vs. 2, 3), Podoplanin avscular (0, 1 vs. 2, 3).

Variable	PT-EPS (n = 28)	cEPS (n = 28)	p
Fibrosis	1/27	2/26	1
FLC	14/14	10/18	0.4
Exudation	12/16	14/14	0.8
Mesothelial denudation	0/28	0/28	1
Acellular areas	16/12	21/7	0.3
Cellularity	12/16	8/20	0.4
Vessel density	9/19	5/23	0.4
Acute inflammation	24/4	23/5	0.3
Chronic inflammation	16/12	13/15	1
Vasculopathy	5/23	6/22	1
Hemorrhage	17/11	15/13	0.8
Fibrin deposits	11/17	10/18	1
Calcification	25/3	26/2	1
Iron deposits	11/17	10/18	1
Ossification	0/0	0/0	1
Podoplanin vascular	16/12	17/11	1
Podoplanin avascular	11/17	10/18	1

Based on the morphological evaluation the biopsies with PT-EPS could not be separated from cEPS (Figure 2). There was no significant difference in any of the scored parameters (Table 2). The prominent staining for podoplanin in EPS biopsies was seen to be identical in both country sources, reproducing previous findings. The scores for podoplanin were not significantly different. The peritoneal biopsies were separated into four groups: “low” podoplanin pattern, “organized” podoplanin pattern, “diffuse” pattern and “mixed” pattern with features of both “organized” and “diffuse” patterns). Using this detailed analysis, no differences could be detected between the PT-EPS and cEPS group (all p>0.05) (table 3). Importantly, the percentages of the various patterns were similar as previously described [31].

**Table 3:** Podoplanin patterns [31] in post-transplantation and classical EPS patients; Organized pattern (0 vs. 1), Diffuse pattern (0 vs. 1), Low pattern (0 vs. 1), Mixed pattern (0 vs. 1).

Variable	Post-transplantation EPS (n = 28)	Classical EPS (n = 28)	p
Organized pattern	10	8	0.8
Diffuse pattern	7	6	1
Low pattern	1	5	0.2
Mixed pattern	4	4	1

To avoid a systematic bias between the peritoneal biopsies from different sources (i.e. the Netherlands and Germany), the results were compared between the biopsies from different countries. There was no statistically significant difference between the peritoneal biopsies from the German patient cohort compared to the patients from the Netherlands (table 4).

**Table 4:** Histological findings in patients with EPS (post-transplantation EPS and classical EPS) in the study population of the Netherlands and Germany; Fibrosis (0, 1 vs. 2, 3), Fibroblast-like-cells (FLC) (0, 1 vs. 2, 3), Exudation (0, 1 vs. 2, 3), Mesothelial denudation (0 vs. 1), Acellular areas (0 vs. 1), Cellularity (0, 1 vs. 2, 3), Vessel density (0, 1 vs. 2, 3), Acute inflammation (0, 1 vs. 2, 3), Chronic inflammation (0, 1 vs. 2, 3), Vasculopathy (0 vs. 1), Hemorrhage (0, 1 vs. 2, 3), Fibrin deposits (0, 1 vs. 2, 3), Calcification (0 vs. 1), Iron deposits (0 vs. 1), Ossification (0 vs. 1), Podoplanin vascular (0, 1 vs. 2, 3), Podoplanin avscular (0, 1 vs. 2, 3).

Variable	Netherlands (n = 18)	Germany (n = 38)	p
Fibrosis	2/16	1/37	0.2
FLC	10/8	14/24	0.3
Exudation	8/10	18/20	1
Mesothelial denudation	0/18	0/38	0.3
Acellular areas	13/5	24/14	0.6
Cellularity	6/12	14/24	1
Vessel density	3/15	11/38	0.5
Acute inflammation	14/4	33/5	0.4
Chronic inflammation	11/7	18/20	0.4
Vasculopathy	5/13	6/32	0.3
Hemorrhage	12/6	20/18	0.8
Fibrin deposits	9/9	12/26	0.2
Calcification	18/0	33/5	0.2
Iron deposits	4/14	17/21	0.1
Ossification	0/0	0/0	1
Podoplanin vascular	13/5	20/18	0.2
Podoplanin avascular	9/9	12/26	0.2

## Discussion

Previous studies suggest, that the clinical course of cEPS differs from patients who develop EPS after transplantation. Therefore, the goal of this study was to compare the morphology of a high number of peritoneal biopsies from patients with PT-EPS and cEPS to detect possible histological differences between the two groups. We matched the groups according to “time on PD”, the most relevant risk factor for the development of EPS. A separation of the two groups on morphological grounds would provide evidence that these reflect two different pathological entities. Inflammation, angiogenesis and fibrosis are the main features of EPS, resulting in exudations of fibrin and chronic inflammation of the peritoneal membrane [35]. This leads to adhesions, development of a fibrous cocoon covering the intestines and results in symptoms of bowel obstruction [10], [36]. The biopsies included in our study demonstrated the typical (but non-specific) morphological features described for EPS. We could not confirm the hypothesis that PT-EPS and cEPS are two different entities, as the histological evaluation could not separate biopsies from the groups. Although, we applied all ever published histological features in EPS, we found no significant differences between the two groups [8]. Immunohistochemstry with podoplanin, including an extensive pattern analysis revealed no difference between the groups. Recently, Kinashi et al. showed that peritoneal tissue from patients with ultrafiltration failure (UFF) contained more lymphatic vessels than tissue from patients without UFF [37]. Podoplanin was found to be a good marker for lymphatic endothelial cells, but is expressed by peritoneal mesothelial and fibroblast-like-cells (FLC) too [8], [38]. In our patient cohort, approximately fifty-percent of the patients in both groups showed a strong expression of podoplanin, but no difference in the expression pattern. Hence, podoplanin seems to play an role in the pathogenesis of EPS, but does not differentiate between cEPS and PT-EPS.

The incidence of EPS increases with time on PD (other factors like peritonitis rate, male gender, younger age, smoking and glucose exposure are under debate) [5], [16], [32], [39]–[44]. The group of patients with cEPS demonstrated a higher-rate of peritonitis, and the use of icodextrin was more common, whereas the time to diagnosis after cessation of PD was longer in the cEPS group. The shorter time to clinically symptomatic EPS in patients after kidney transplantation has previously been described [21], [45]. Interestingly, there were no differences regarding morphological findings, and particularly no differences in the severity of fibrosis, using both, semi-quantitative and quantitative analysis. This could argue that factors in the PT-EPS group resulted in a faster progression of the disease. It is likely that the time to the clinical manifestation is based on the ratio of pro fibrotic factors (e.g. time on PD, surgery, peritonitis, calcineurin-inhibitors) and factors which might inhibit the disease process (e.g. steroids, rinsing the abdominal cavity). The combination of pro-fibrotic factors after transplantation might result in a faster disease process, although the major players in the pathogenesis have not been defined [21].

It has been recently shown in a rat model of peritoneal exposure to dialysis fluid that additional administration of Ciclosporin leads to EPS like abnormalities [46]. The calcineurin-inhibitors (Ciclopsorin and Tacrolimus) can lead to enhanced expression of transforming growth factor-β (TGF-β), demonstrated in a preconditioned peritoneal membrane, which already demonstrated up-regulation of TGF-β. This results in increasing fibrosis and neoangiogenesis of the peritoneal membrane [21], [26], [47]. Due to the lack of biopsies at the time of transfer to either dialysis or transplantation we cannot prove the differences in progression. It is less likely that PT-EPS patients would have had more severe membrane injury at the time of modality transfer compared to the cEPS group who likely had complications leading to technique failure as evidenced by their higher rate of peritonitis episodes and greater need of Icodextrin, suggesting either UFF or less residual renal function. This raises an important question: could the morphological evaluation at the time of transfer to a different form of renal replacement therapy add value to an overall risk assessment (including time on treatment, peritonitis history and ultrafiltration failure) in predicting the development of EPS? This requires a high number of patients with biopsies at transfer when removing the PD catheter, and we will try to answer this question within the European patient cohort in the future.

An alternative explanation for the differences between the time to manifestation would be that patients after transplantation might be seen more often in the transplant clinics and/or symptoms might rather be ignored in patients on hemodialysis, as had been reported by patients [48]. In 9 interviews of patients with EPS the patients described a loss of trust in the doctors as symptoms while being on dialysis were not taken seriously [48].

If patients after transplantation would be detected earlier in the diseases course due to more frequent doctor visits, it would be expected that the histological finding would be less extensive in patients with PT-EPS. Particularly, the fibrosis scores should be less severe, but no differences could be detected and time from onset of symptoms associated with EPS to requirement of surgery was not different between both groups. Therefore this argument is less convincing than a faster disease process.

The limitations of the study design of course leaves unanswered questions and room for future studies. The available data from these two referral centers provided in the registers were limited. We could not provide more sophisticated information about membrane function (e.g. osmotic conductance or glucose exposure during PD) [49]. We were unable to fully comply with suggested standards for reporting clinical features of EPS as the data was collected prospectively, before the standards were suggested [50]. Survival was not significantly different between the groups, even so there was a trend towards a better outcome in the PT-EPS group (p = 0.3). This does not contradict previous reports, which demonstrated a better outcome of PT-EPS, as the study was not powered to find such a difference [19], [45]. The younger age of PT-EPS patients and the better overall condition of patients with a functioning kidney allograft would be likely explanations for a better outcome [19].

In conclusion, this analysis did not support the hypothesis that EPS following transplantation is a different clinico-pathological entity, despite differences in the time taken between stopping PD and diagnosis and possible differences in known risk factors such membrane failure.
